# Utilization of a Digital Radiographic Imaging System for Assessing Ventilation and Guiding Rehabilitation in a Post-lung Transplant Patient: A Case Report

**DOI:** 10.7759/cureus.87955

**Published:** 2025-07-14

**Authors:** Akihiro Kanaya, Shoma Tanaka, Hidenobu Takagi, Elvedin Lukovic, Masanori Yamauchi

**Affiliations:** 1 Department of Anesthesiology, Tohoku University Hospital, Sendai, JPN; 2 Department of Radiology, Tohoku University Hospital, Sendai, JPN; 3 Department of Anesthesiology, Vagelos College of Physicians and Surgeons, Columbia University, New York, USA

**Keywords:** digital radiographic imaging system, dyspnea, fluid management, lung transplantation, rehabilitation, ventilatory assessment

## Abstract

Following lung transplantation, patients frequently experience dyspnea due to ischemia-reperfusion injury, pulmonary edema, vagotomy, and diaphragmatic nerve paralysis. Dynamic digital radiography (DDR), a digital radiographic imaging system, provides dynamic visualization of lung ventilation, facilitating targeted interventions. We report a case of a patient with persistent post-transplant dyspnea who exhibited significant improvement following active rehabilitation and fluid management, guided by DDR assessments. DDR may serve as a valuable tool for early post-transplant management and long-term pulmonary monitoring.

## Introduction

Dyspnea, a common complication following lung transplantation, frequently results from ischemia-reperfusion injury, pulmonary edema, diaphragmatic dysfunction, or factors such as preoperative respiratory failure and postoperative pain [1]. Accurate assessment of lung ventilation and diaphragmatic motion is required for effective post-transplant management. Conventional imaging modalities, including chest X-ray and computed tomography (CT), offer only static snapshots of pulmonary function and may not completely capture dynamic ventilation abnormalities. Dynamic digital radiography (DDR), an advanced imaging technique, facilitates real-time lung function evaluation by capturing continuous respiratory motion sequences [2]. To improve patient outcomes following lung transplantation, the factors influencing dyspnea and its management should be elucidated. This case report highlights the utility of DDR in detecting hypoventilated regions and diaphragmatic motions, thereby guiding targeted rehabilitation strategies and optimizing fluid management in a lung transplant recipient.

## Case presentation

**Figure 1:**
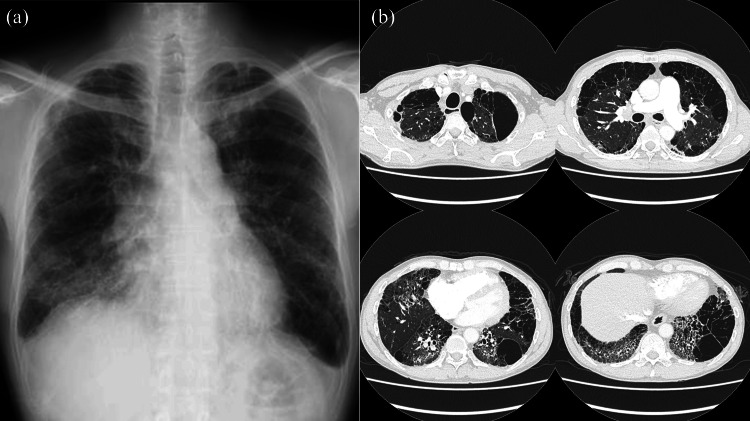
Patient’s chest X-ray (a) and computed tomography (CT) (b) images before bilateral lung transplantation Chest X-ray reveals lower lobe reticulation. High-resolution CT shows upper lobe emphysema and lower lobe honeycombing and traction bronchiectasis.

A 50-year-old male patient was listed for cadaveric bilateral lung transplantation (BLTx) for end-stage interstitial pneumonia secondary to systemic scleroderma. His respiratory function was moderately impaired, and echocardiography revealed secondary pulmonary hypertension (Table 1). Chest X-ray and CT revealed upper lobe emphysema and lower lobe honeycombing and traction bronchiectasis (Figure 1). He underwent BLTx under veno-arterial extracorporeal membranous oxygenation support. Blood loss during the BLTx was 2,009 mL. His chest was closed during the first surgery as lung edema did not develop.

**Table 1:** Spirometric and echocardiographic data of the patient before BLTx BLTx: bilateral lung transplantation; FEV1.0: forced expiratory volume in one second; FVC: forced vital capacity; LVEDd: left ventricular end-diastolic diameter; LVEF: left ventricular ejection fraction; TR: tricuspid valve regurgitation; TRPG: maximum tricuspid regurgitation pressure gradient; %FEV1.0: percent-predicted FEV1.0; %FVC: percent-predicted FVC

		Reference range
Spirometry		
FVC (mL)	3,050	-
%FVC (%)	80.0	>80
FEV1.0 (mL)	2,470	-
%FEV1.0 (%)	81.0	>70
Echocardiography		
LVEDd (mm)	42	41-52
LVEF (%)	68	59-71
TRPG (mmHg)	69	<35
Others	TR Ⅰ°	-

**Figure 2:**
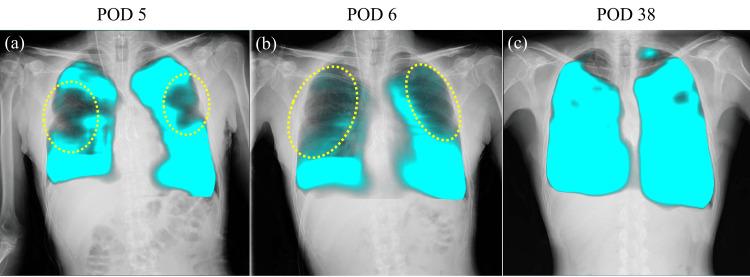
Patient’s dynamic images obtained using dynamic digital radiography (DDR) (PL-mode) In the PL-mode, the dynamic ventilation image is shown, demonstrating a blue shadow in the position where the lung tissue is expanded by breathing. Yellow dotted ellipses indicate hypoventilation. PL-mode: pixel value measurement in a low-frequency mode

**Figure 3:**
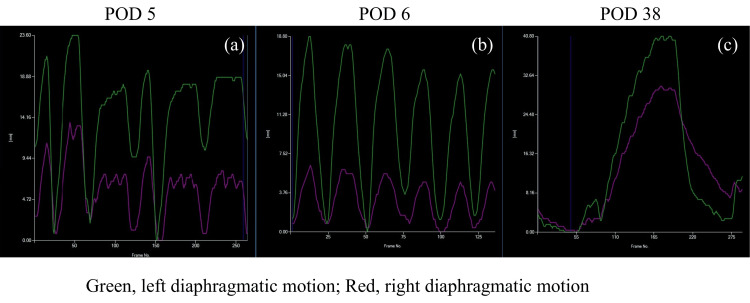
Patient’s diaphragmatic motion assessed using dynamic digital radiography (DDR) (DM-mode) observed, the right diaphragm exhibits a weaker motion than the left side. Vertical axis: Diaphragm displacement distance. Horizontal axis: Time. DM-mode: diaphragm movement mode

Despite stable blood gas parameters and absence of respiratory acidosis, the patient experienced persistent dyspnea and tachypnea, necessitating extended ventilator support. The respiratory rate ranged from 20 to 30 breaths per minute. On postoperative day (POD) 5, DDR was performed to evaluate lung and diaphragmatic motions. DDR was performed using a conventional radiography system (RADSpeed Pro, Shimadzu, Kyoto, Japan) and a flat-panel detector (AeroDR fine, Konica Minolta, Tokyo, Japan). The image data set was analyzed using dedicated software (KINOSIS, Konica Minolta, Tokyo, Japan). DDR, assessed using the pixel value measurement in a low-frequency mode demonstrating a blue shadow in the position where the lung tissue is expanded by breathing, revealed reduced ventilation in the middle lung field (Figure 2a: corresponding video materials are available in Video 1a). The diaphragm movement mode tracks the diaphragm’s movement across sequential frames, measures its vertical displacement, and presents the data graphically. Although findings suggestive of phrenic nerve palsy were not observed, the right diaphragm demonstrated a weaker motion than the left side (Figure 3a).

**Video 1:** Dynamic images obtained by dynamic digital radiography (DDR) (PL-MODE) PL-mode: pixel value measurement in a low-frequency mode

On POD 6, the patient was extubated as his respiratory pattern improved, with decreasing tachypnea. However, post-extubation DDR revealed worsened ventilation in the upper and middle lung regions (Figure 2b: corresponding video materials are available in Video 1b). Furthermore, DDR showed more impaired motion of the right diaphragm compared to the left on POD6; however, no obvious phrenic nerve palsy was noted (Figure 3b). Physical limitations such as chest drains and postoperative pain made it difficult to perform ultrasound assessments. As diaphragmatic motion had already been assessed using DDR, an additional ultrasound examination was not needed. As ventilator support discontinuation caused the worsening of these symptoms (tachypnea and dyspnea), aggressive rehabilitation, including deep breathing exercises, limb rehabilitation, sitting, and walking, along with dry-side fluid management, was implemented. On POD 9, he was discharged from the intensive care unit as his dyspnea and tachypnea gradually improved. On POD 38, follow-up DDR revealed improved ventilation (Figure 2c: corresponding video materials are available in Video 1c) and diaphragmatic motion (Figure 3C). On POD 48, the patient was successfully discharged.

## Discussion

Post-lung transplantation dyspnea is a complex issue affected by several factors, including ischemia-reperfusion injury, pulmonary edema, vagotomy, and diaphragmatic dysfunction. Conventional static imaging modalities, including chest X-ray and CT, offer limited insight into dynamic pulmonary mechanics. In contrast, DDR provides a distinct advantage by capturing real-time respiratory motion, enabling a more comprehensive evaluation of ventilation patterns and diaphragmatic function. In this case, DDR contributed to identifying hypoventilated lung regions and detecting asymmetrical diaphragmatic movements. This real-time feedback enabled clinicians to personalize the patient’s rehabilitation plan, underscoring diaphragmatic strengthening exercises, such as deep breathing, and optimizing fluid management strategies for pulmonary congestion prevention.

DDR successfully delineated post-transplant hypoventilation and diaphragmatic motion abnormalities undetectable via conventional imaging. On POD 5, despite the absence of obvious radiographic evidence of phrenic nerve palsy, DDR revealed reduced middle lung field ventilation and attenuated right diaphragmatic motion. These findings suggest a transient functional impairment rather than structural pathology, corroborating prior studies indicating that post-transplantation diaphragmatic dysfunction can result from intraoperative factors, including intraoperative handling or ischemia [3].

Following extubation on POD 6, DDR demonstrated further deterioration in ventilation, particularly in the upper and middle lung fields, as well as progressive right diaphragmatic motion weakening, which may be attributed to ventilator support discontinuation. Therefore, rehabilitation and dry-side fluid management were continued. On POD 38, DDR revealed enhanced ventilation and diaphragmatic motion, paralleling the patient’s symptomatic relief and facilitating hospital discharge on POD 48. These findings highlight the utility of DDR in the longitudinal assessment of pulmonary mechanics, guiding real-time therapeutic adjustments and optimizing post-transplant recovery.

Rehabilitation plays a significant role in post-lung transplantation management, particularly for patients experiencing ventilatory insufficiency and diaphragmatic dysfunction. Studies have reported that early and intensive rehabilitation, including deep breathing exercises, limb rehabilitation, sitting, and walking, improves lung function and reduces the risk of postoperative complications [4]. Mobilization strategies help prevent atelectasis, improve diaphragmatic motion, and enhance oxygenation, contributing to overall respiratory recovery [5]. The patient exhibited progressive symptom improvement with a rehabilitation program integrating these interventions.

DDR provides benefits in assessing diaphragm mechanics, which are critical for post-transplant recovery. Phrenic nerve injury, surgical manipulation, or prolonged mechanical ventilation can cause diaphragmatic dysfunction following lung transplantation. Although phrenic nerve palsy frequently manifests with paradoxical diaphragmatic movement on fluoroscopy or ultrasonography, milder dysfunctions may be more challenging to detect. Compared to ultrasound, DDR may be more sensitive in detecting mild diaphragmatic dysfunction. This is because ultrasound is operator-dependent and limited by acoustic windows, body habitus, and patient cooperation. In contrast, DDR provides a dynamic, full-field visualization of both hemidiaphragms simultaneously over the entire respiratory cycle, enabling the detection of subtle motion abnormalities that may be missed by point-based ultrasound assessment. DDR, with its ability to dynamically quantify diaphragmatic excursion, offers an additional tool to detect diaphragmatic impairment early and monitor progress over time [6]. The gradual improvement in the right diaphragmatic movement in our patient supports the hypothesis that the dysfunction was transient and associated with perioperative factors rather than permanent nerve injury.

From a practical perspective, DDR does not require contrast administration and can be performed using conventional digital radiography systems available in most healthcare facilities. Compared with dynamic fluoroscopy, DDR offers the benefit of reduced radiation exposure while still capturing high-temporal-resolution imaging of lung function [7,8]. These attributes make DDR a promising adjunctive tool for routine post-transplant surveillance, particularly in patients with persistent or unexplained respiratory symptoms.

## Conclusions

In conclusion, this case highlights the utility of DDR in assessing post-lung transplant ventilation abnormalities and diaphragmatic dysfunction, enabling rehabilitation and fluid management approaches. DDR provided real-time insights into pulmonary mechanics, facilitating timely therapeutic adjustments that contributed to the patient’s successful recovery. Considering low radiation exposure and the ability to dynamically evaluate lung function, DDR may serve as a valuable tool for both early post-transplant management and long-term pulmonary monitoring. To validate the role of DDR in predicting long-term transplant outcomes and optimizing postoperative management strategies, further studies are warranted.
